# Dislodgement of a subcutaneous shock lead owing to loop release in the device pocket in a pediatric patient with congenital long QT syndrome

**DOI:** 10.1016/j.hrcr.2026.02.006

**Published:** 2026-02-14

**Authors:** Yoshiaki Kato, Heima Sakaguchi, Yuji Tominaga, Kazuki Matsubara, Shigemitsu Iwai, Kenichi Kurosaki

**Affiliations:** 1 Department of Pediatric Cardiology, National Cerebral and Cardiovascular Center, Osaka, Japan; 2 Department of Pediatric Cardiovascular Surgery, National Cerebral and Cardiovascular Center, Osaka, Japan

**Keywords:** Pediatric ICD, Subcutaneous shock lead, Lead dislodgement, Long QT syndrome, Device pocket loop, Spiral spring

**Key Teaching Points**

• Shock lead placement in implantable cardioverter-defibrillators in small infants requires tailored approaches for each case.
• Lead dislodgement may occur through loop loosening within the generator pocket, a mechanism distinct from that of Twiddler syndrome, Reel syndrome, or Ratchet syndrome.
• Careful adjustment of the lead-loop configuration relative to the pocket size at implantation is essential to prevent traction-related complications.

## Introduction

Implantable cardioverter-defibrillators (ICDs) are increasingly used in pediatric patients for both primary and secondary prevention of sudden cardiac death. In this population, ICD management requires consideration of small body size, growth-related changes, and disease-specific characteristics. In infants and young children, conventional transvenous lead placement for ICD implantation is often challenging because of the small vascular caliber, and alternative strategies—such as positioning shock leads in subcutaneous or epicardial locations—are employed on a case-by-case basis.^1^^,^^2^ We present a rare case of dislodgement of a subcutaneous shock lead because of loop release within the device pocket in a pediatric patient with congenital long QT syndrome.

## Case report

**Figure 1:**
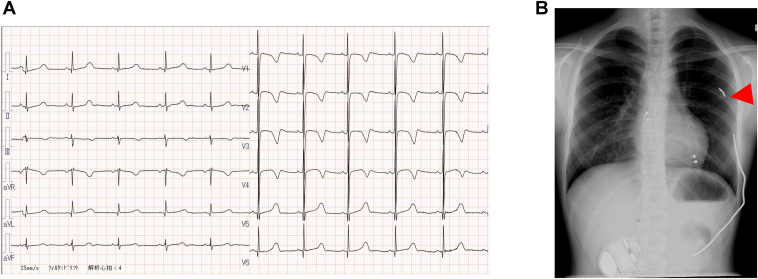
A 12-lead ECG obtained at the age of three years showed QT prolongation (**A**). Chest and abdominal radiograph obtained at 13 years of age, prior to the generator replacement and lead revision, showed complete detachment of the distal tip of the subcutaneous shock lead (*red arrowhead*) (**B**). ECG = electrocardiogram.

The patient was a 13-year-old girl with long QT syndrome (LQTS) who underwent replacement of her implantable cardioverter-defibrillator (ICD) generator and subcutaneous shock lead. The patient had no family history of sudden cardiac death. At age 1, the patient experienced ventricular fibrillation during exertion, requiring cardiopulmonary resuscitation and automated external defibrillator shocks. LQTS was diagnosed on the basis of electrocardiographic findings, and genetic testing was performed; however, no pathogenic variants were identified. At the age of 4 years, with a height of 108 cm and a weight of 17.6 kg, the patient underwent ICD implantation for secondary prevention. There was a 2.5-year interval between the ventricular fibrillation event and ICD implantation. Initially, the family elected continuous supervision while carrying an automated external defibrillator; as this approach became increasingly impractical, ICD implantation was reconsidered approximately 2 years later. This prompted repeated discussions among the family, the previous attending physician, and our team. Although no cardiac events had occurred during the preceding 2 years on propranolol, future risk could not be excluded; therefore, after a thorough risk–benefit review and in accordance with the family’s preference, we proceeded with ICD implantation. Pacing was deemed necessary in this case to allow further up-titration of β-blocker therapy and to prevent bradycardia-induced Torsades de Pointes. Owing to the patient’s small body size (108 cm, 17.6 kg), a transvenous lead system was deemed inappropriate. Consequently, a thoracotomy was performed, and atrial and ventricular epicardial pacing leads were placed. In addition, a 45-cm subcutaneous shock lead (model 6996SQ, Medtronic) was implanted subcutaneously with the distal tip positioned posteriorly, and connected to an abdominal generator placed in a post-rectus pocket. The patient subsequently remained free of ICD shocks, with nadolol therapy and atrial pacing. With somatic growth, the shock lead was progressively stretched, ultimately resulting in a fracture at its distal tip (Figure 1B). Owing to the structural characteristics of the subcutaneous shock lead, the tip does not function as an electrode. Therefore, even if the tip becomes disconnected, shock delivery remains effective as long as the shock coil remains intact. For this reason, the lead was left in place and continued to be used. At age 13 years, generator replacement was required owing to progressive battery depletion; although conversion to a transvenous lead system was feasible, concerns regarding venous occlusion and other long-term complications supported replacement of the subcutaneous shock lead.

**Figure 2:**
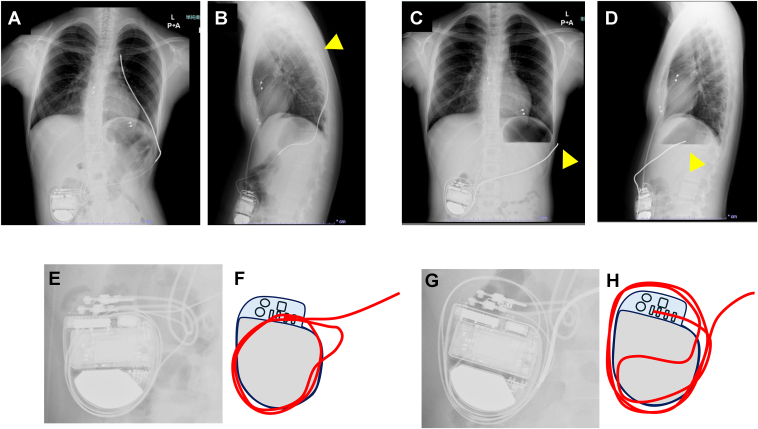
Chest and abdominal radiographs. The left panels (**A, B, E, F**) were obtained immediately after surgery, and the right panels (**C, D, G, H**) after lead dislodgement. The upper row shows the frontal (**A, C**) and lateral (**B, D**) views, with *arrowheads* indicating the tip of the shock lead. The lower row shows magnified frontal views (**E, H**) demonstrating the positional relationship between the generator and the leads, with the shock lead highlighted in *red* in the schematic illustrations (**F, H**). Although the positions of the generator and pacing lead remained unchanged, the loop configuration of the shock lead exhibited a marked alteration.

The height of the patient at the time of the operation was 153.6 cm, and the weight was 45 kg. Intra-operatively, an 85-cm subcutaneous shock lead was tunneled and sutured using an anchoring sleeve to the fascia at the left thoracic incision. Excess lead was looped and stored within the pocket (Figure 2, left panels). The immediate post-operative course was uneventful. However, 50 days post-operatively, in the absence of any identifiable trigger, remote monitoring revealed a sudden drop in lead impedance, coinciding with the patient’s acute onset of pain localized to the lead position in the left abdominal region. Chest radiography (Figure 2, right panels) revealed retraction of the shock lead tip toward the lateral abdominal region, with no positional change of the generator or pacing leads. The loop configuration of the shock lead within the pocket had altered, with a previously small loop becoming enlarged and the lead being pulled inward. As the device could no longer function appropriately as an ICD, the defibrillation capability was deactivated, and the patient has since been managed with atrial pacing. At present, the patient and her family are being consulted regarding subsequent management options for the ICD.

## Discussion

**Figure 3:**
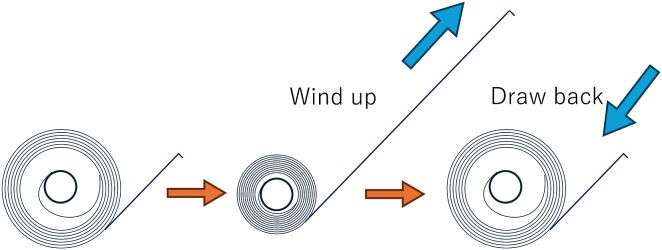
Schematic illustration of a spiral torsion spring mechanism. The formation of a small loop of the lead within the pocket created a state analogous to the winding of a measuring tape spring, in which potential energy was stored in the coil. The subsequent restoring force of the spring exerted a retractive pull, resulting in the lead being drawn back.

**Figure 4:**
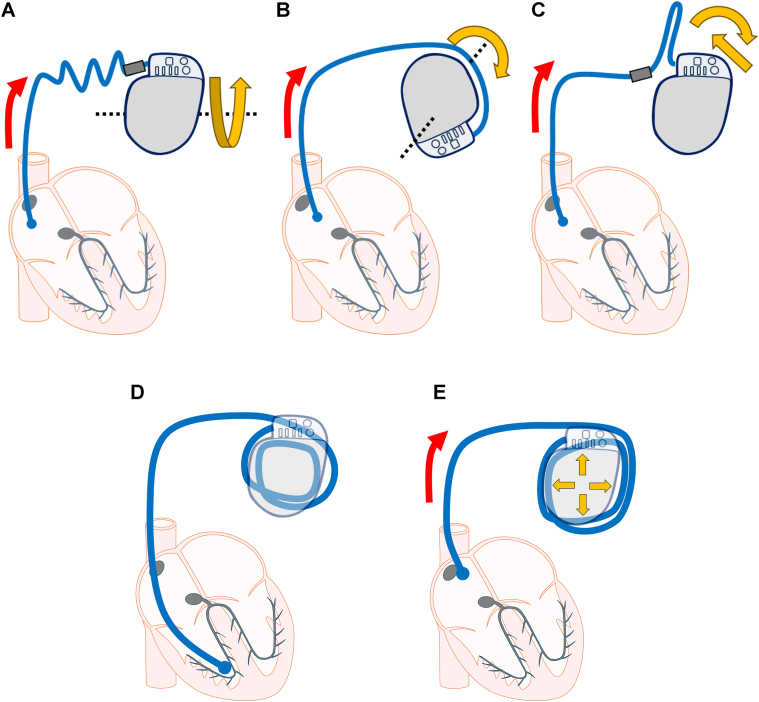
Schematic illustration of the mechanisms of lead dislodgement: (**A**) Twiddler’s syndrome, where generator rotation along its longitudinal axis results in lead traction and twisting. (**B**) Reel syndrome, in which generator rotation along its transverse axis causes the lead to be drawn into the pocket. (**C**) Ratchet syndrome, where generator displacement owing to arm movement produces lead traction, limited by the anchoring sleeve so that traction occurs only on the generator side of the sleeve. (**D**) and (**E**) The mechanism observed in the present case using a schematic of a transvenous lead: the lead was initially accommodated within the pocket as small loops (**D**), but when these loops suddenly loosened into larger loops, the lead was drawn further into the pocket (**E**).

This case illustrates a unique mechanism of subcutaneous shock lead dislodgement in a pediatric patient. The unwinding of a stored lead loop within the device pocket can generate retractive forces, similar to a spiral torsion spring, leading to proximal migration of the lead (Figure 3). Although Twiddler, Reel, and Ratchet syndromes are recognized causes of lead dislodgement,^3^ this mechanism observed in this case differed in that it occurred without generator rotation or patient manipulation (Figure 4). Potential contributing factors in this case included a mismatch between the pocket size and the lead loop. Although it is common practice to accommodate excess lead within the pocket by forming a loop, in this case, the presence of a pre-existing pacing lead, whose position was essentially fixed, made it difficult to rotate the generator to adjust for the excess lead as described in the product manual. Consequently, only a relatively small loop could be created within the pocket, which was considered to have generated a spring-like retractive force. In retrospect, greater attention to optimizing the balance between loop size and pocket dimensions, together with more secure fixation of the anchoring sleeve, might have prevented this complication.

With regard to the subcutaneous shock lead, the manufacturer, Medtronic, has announced the discontinuation of the 41-cm model, which has occasionally been used in small pediatric patients. In such patients, the use of longer subcutaneous shock leads often necessitates the creation of loops within the pocket, and therefore, physicians caring for pediatric patients should be aware of the potential complications associated with this practice. Moreover, this phenomenon is not limited to subcutaneous shock leads; similar issues may also arise in implantable devices using conventional transvenous leads, particularly in ICD leads with strong elastic properties, when excess lead length is accommodated by loop formation, warranting careful attention.

## Conclusion

In conclusion, this case illustrates that when excess lead length must be accommodated within a pocket by creating loops, careful adjustment of the loop configuration relative to the pocket size is essential to prevent excessive traction and potential complications. It also underscores the importance of securing the lead adequately.

## Disclosures

The authors have no conflicts of interest to disclose.
